# *Arabidopsis* floral phytomer development: auxin response relative to biphasic modes of organ initiation

**DOI:** 10.1093/jxb/eru153

**Published:** 2014-04-17

**Authors:** J. W. Chandler, W. Werr

**Affiliations:** ^1^Institute of Developmental Biology, Cologne Biocenter, University of Cologne, Cologne, Germany.

**Keywords:** Centripetal flower development, *DORNRÖSCHEN-LIKE*, stem-cell niche, founder cells, floral meristem, bract.

## Abstract

In the *Arabidopsis* inflorescence meristem (IM), auxin is considered a prepatterning signal for floral primordia, whereas a centripetal mode of positional information for floral organ identity is inherent to the ABCE model. However, spatio-temporal patterns of organ initiation in each whorl at the earliest initiation stages are largely unknown. Evidence suggests that initial flower development occurs along an abaxial/adaxial axis and conforms to phytomer theory. Use of the founder cell marker *DORNRÖSCHEN-LIKE* (*DRNL*) as a tool in *leafy*, *puchi*, and *apetala 1 cauliflower* mutant backgrounds suggests that bract founder cells are marked at the IM periphery. The *DRNL* transcription domain in the wild-type IM is spatially discrete from *DR5* expression, suggesting that bract initiation is independent of canonical auxin response. When bracts develop in *lfy* and *puchi* mutant floral primordia the initiation of lateral sepals precedes the specification of medial sepals compared with wild type, showing an interplay between bract and abaxial sepal founder cell recruitment. In the *perianthia* (*pan*) mutant background, *DRNL* expression indicates that a radial outer whorl arrangement derives from splitting of sepal founder cell populations at abaxial and adaxial positions. This splitting of incipient sepal primordia is partially dependent on *PRESSED FLOWER* (*PRS*) activity and implies that sepal specification is independent of *WUSCHEL* and *CLAVATA3* expression, as both marker genes only regain activity in stage-2 flowers, when patterning of inner floral organs switches to a centripetal mode. The transition from an initially abaxial/adaxial into a centripetal patterning programme, and its timing represent an adaptive trait that possibly contributes to variation in floral morphology, especially unidirectional organ initiation.

## Summary

A discrepancy exists between auxin response maxima and floral organ initiation, which occurs unidirectionally for sepals and transitions to a centripetal mode for inner whorl organs.

## Introduction

Angiosperm flowers are comprised of concentric whorls of outer perianth and inner androecium organs and a central gynoecium. Positional information that determines the identity of floral organs in each whorl is embodied by the iconic centro-radial ABCE model, based on the dicot species *Arabidopsis thaliana* and *Antirrhinum majus*, where floral organ differentiation is choreographed by transcriptional regulation by mostly MADS-box transcription factors (Coen and Meyerowitz, 1991; Theissen and Saedler, 2001). The ABCE model is broadly conserved among eudicots (Kanno *et al.*, 2003) and monocots (Whipple *et al.*, 2004), with modifications among basal angiosperms (Soltis *et al.*, 2007). However, it only accounts for organ identity, and not the relative timing of organ initiation or positions within whorls. Homeotic genes function later than organ initiation, and the two processes can be genetically uncoupled (Bowman *et al.*, 1989; Hill and Lord, 1989; Crone and Lord, 1994). The earliest organogenesis events comprise the perception of positional signals by a single cell or groups of cells on the flank of the inflorescence meristem (IM) or floral meristem (FM), which causes fate specification, and subsequent activation results in controlled cell proliferation to generate an anlage or preprimodium, and then a histologically visible primordium (Beveridge *et al.*, 2007; Chandler, 2011). In *Arabidopsis*, medial stamens become morphologically evident slightly before petals and lateral stamens (Smyth *et al.*, 1990). Therefore, to analyse whether the pattern of organ founder cell specification in the FM is reflected by the sequence of organ primordia appearance is an important question that has been hampered to date by the lack of organ founder cell markers. Despite the ubiquity of a centripetal model paradigm in angiosperms, there are many examples of alternative modes of floral architecture establishment. For example, many of over 300 analysed legume species show unidirectional floral organ initiation along an abaxial/adaxial axis or a helical or bidirectional initiation (Tucker, 2000, 2001, 2003), coupled with overlapping temporal organ initiation in different whorls and common floral organ primordia, such as *Pisum sativum* (Ferrándiz *et al.* 1999) or *Medicago truncatula* (Benlloch *et al.*, 2003). In other species, organ development within a single flower or whorl can show centripetal, centrifugal (or basipetal), or more chaotic sequences (Rudall, 2010). These floral body plans highlight that a single unifying centripetal model of organ initiation is open to challenge.

Evidence from *Arabidopsis* suggests that floral organ initiation begins along an abaxial/adaxial axis. Firstly, the FM does not simply separate from the IM, but conforms to phytomer theory, forming in the axil of a cryptic bract that emerges at the IM periphery during floral stage 1 and whose outgrowth is suppressed (Kwiatkowska, 2006; Alvarez-Buylla *et al.*, 2010). Bract initiation followed by FM initiation on its adaxial flank establishes an abaxial/adaxial axis of development. Secondly, during stages 1 and 2, expression of *DORNRÖSCHEN-LIKE* (*DRNL*), an AP2 transcription factor and founder cell marker, marks each sepal unidirectionally in the order: abaxial, simultaneously two lateral, adaxial (Chandler *et al.*, 2011). The specification of sepal founder cells occurs in the absence of expression of the *CLAVATA3* (*CLV3*) and *WUSCHEL* (*WUS*) stem-cell markers, which mark the IM stem cell population (Fletcher *et al.* 1999; Laux *et al.* 1996) but are absent at the IM periphery until the floral buttress has completely separated from the IM, and reappear in the centre of the FM at late floral stage 2 (Goldschmidt *et al.*, 2008; Yadav *et al.*, 2009). This discontinuity in *CLV3/WUS* expression coincides with the sequential initiation of all four sepals. Thirdly, the elaboration of the lateral sepals strictly depends on the activity of *PRESSED FLOWER*, a close WUS relative that promotes cell proliferation in the lateral floral primordium domains (Matsumoto and Okada, 2001). Fourthly, mutants in the *PUCHI* transcription factor show a mosaic floral organ/inflorescence phenotype, where floral meristems emerge from the IM and partially differentiate along an abaxial/adaxial axis, before reverting to IMs (Karim *et al.*, 2009). Finally, based on conceptual homology between flowers and leaves as lateral organs, the conservation of polarity genes such as *YABBY* transcription factors, including *FILAMENTOUS FLOWER* in flowers (Sawa *et al.*, 1999; Eshed *et al.*, 2001; Lugassi *et al.*, 2010), suggests they might perform a similar role in the FM. *FILAMENTOUS FLOWER* is expressed polarly in stage-1 flowers and leads to a strong mutant floral phenotype, supporting the hypothesis that abaxial/adaxial polarity is the dominant developmental mode in the early FM. These data consistently imply that patterning of the outer sepal whorl in *Arabidopsis* is not a centroradial process, but follows an abaxial/adaxial gradient, which in the absence of *WUS* or *CLV3* expression, precedes the acquisition of FM autonomy.

Following the re-establishment of a stem cell population in late stage-2 flowers, lateral fields of *DRNL* expression prepattern petal and lateral stamen founder cells, and *DRNL* expression at later stages is compatible with a centripetal mode of organ initiation: a central ring-shaped domain subsequently resolves into four medial stamens, before expression marks the carpels (Chandler *et al.*, 2011).

Prepatterning models suggest that positional signals for all organ whorls are perceived within the FM before organ formation, and the coincidence of auxin maxima with lateral organ initiation in the SAM and IM and the absence of floral organs in auxin-deficient mutants (Cheng *et al.*, 2006) imply that auxin is one instructive signal. However, auxin efflux transport and corresponding response maxima occur in the epidermis (Reinhardt *et al.*, 2003), whereas initial floral organogenesis events are observed in underlying layers, where, for example, *Arabidopsis* petals originate from a periclinal division in the L2 (Hill and Lord, 1989). Also, the timing of auxin response maxima revealed by *DR5* activity associate auxin response more with organ outgrowth in the FM than with initiation (Aloni *et al.*, 2006). Thus, the molecular basis of positional information within the FM that leads to the correct positioning and number of floral organs is poorly understood. Furthermore, unidirectional sepal development, the discontinuity of the stem cell niche in the early FM, and the centripetal model of organ initiation in the inner whorls suggest that both the implementation and the perception of positional information are dynamic and have differing mechanisms.

Mutant backgrounds that are perturbed in floral body plan organization or meristem identity are instructive to address early events in floral patterning in *Arabidopsis*. Mutation of the *PERIANTHIA* (*PAN*) b-ZIP transcription factor induces an apparent radial symmetry; *pan* mutants have increased organ numbers in whorls 1 and 2 and fewer stamens, so that flowers are basically pentameric (Running and Meyerowitz, 1996). The *leafy* (*lfy*) mutant is impaired in FM identity and has bracts (Schultz and Haughn, 1991; Weigel *et al.*, 1992), as does the *puchi* mutant (Karim *et al.*, 2009), whereas the *apetala1 cauliflower* (*ap1 cal*) double mutant proliferates IMs or FMs that are arrested at early developmental stages before organ initiation (Ferrándiz *et al.* 2000).

The aim of this study was to use *DRNL* as a founder cell marker in these mutant backgrounds and to establish the pattern of founder cell initiation at a fine temporo-spatial resolution; special emphasis was placed on: (i) the identity of the founder cells marked by *DRNL* at the IM periphery; (ii) the relationship between bract and sepal initiation; (iii) the correlation between founder cell populations and auxin response maxima; (iv) the origin of supernumerary organs in *pan* mutant flowers. Our data show that the earliest foci of *DRNL* expression in the IM of *lfy* and *puchi* mutants represent bract founder cells. The similar domain in wild type is specified at the outer periphery of canonical auxin response maxima. Bract development in *puchi* or *leafy* mutants flowers alters the order of outer whorl organ initiation and suggests competition between bract and abaxial sepal fate. In contrast, additional sepals in the *pan* mutant arise from a splitting of the abaxial or adaxial sepal founder cell populations through a novel organogenesis mechanism that genetically involves *PRS* function. The dominance of initial abaxial/adaxial patterning even in radial floral body plans has intriguing consequences in an evolutionary developmental context.

## Materials and methods

### Plant material and growth conditions

Plants were grown on soil in the greenhouse in long-day conditions (16h light:8h dark). The *DR5::GFP* line was a gift from J. Friml (VIB, Ghent, Belgium); *DR5::NLS3*×*VENUS* was provided by J.Traas (INRA, CNRS, ENS, Lyon University, France); the *prs* mutant was obtained from M. Scanlon (Cornell University, USA) and the *pan-1* mutant was provided by F. Besnard (INRA, CNRS, ENS, Lyon University, France). The *lfy-1* allele and *ap1-1 cal-1* mutants were obtained from NASC (N6228 and N6161, respectively, Nottingham, UK) and the *puchi* mutant was obtained from M. Tasaka (Nara Institute of Science and Technology, Takayama, Japan). The *drnl-1* allele (Chandler *et al.*, 2007) was used for all experiments. Floral organ phenotypes were described using approximately the first ten flowers from an inflorescence. The *DRNL::erGFP* and *DRNL::CERULEAN* transgenic lines have been described in Chandler *et al.* (2011) and Cole *et al.* (2013), respectively.

### Statistical analyses

Differences in mean floral organ numbers were analysed for significance using unpaired students *t*-tests. Correlations between the numbers of floral organs were tested using Pearson’s correlation coefficient.

### Confocal imaging

Live imaging of GFP, CERULEAN or VENUS fluorophores was performed with a Zeiss LSM 700 confocal laser-scanning microscope (CLSM). GFP was excited at 488nm and emission analysed between 490 and 560nm. CERULEAN excitation and emission wavelengths were 405 and 390–460nm, respectively. To minimise VENUS (yellow) fluorescence in the GFP (green) channel, VENUS was excited with a 514nm laser and emission detected between 525nm and 570nm. For depiction of inflorescence/floral structures, material was excited at 639nm and emission analysed between 640 and 700nm to visualise chlorophyll autofluorescence. Floral staging was defined according to Smyth *et al.* (1990). CLSM images were processed using Photoshop CS2 software (Adobe) and Z-stacks were converted into 3D images using Imaris software (Bitplane, Zürich, Switzerland).

### Sequencing and genotyping

To identify homozygous *pan-1* plants, genomic DNA was subjected to PCR with the primers PANF (5′-TTCACTCCTGATT TCTACTCT-3′) and PANR (5′-GCGAGCTCTTTTGAGCTCTT C-3′), which span the point mutation in *pan-1* (Chuang *et al.*, 1999) and produce a product of 603bp. PCR amplicons were sequenced with the primer PANseq (5′-GGTGGCTTGAGGGAGAGACTT-3′). The homozygous wild-type locus could be distinguished from that of homozygous mutant and heterozygous plants by the nucleotide peaks.

## Results

### The initiation of lateral organ founder cells in the IM relates to, but does not correlate with, auxin response maxima

**Fig. 1.**
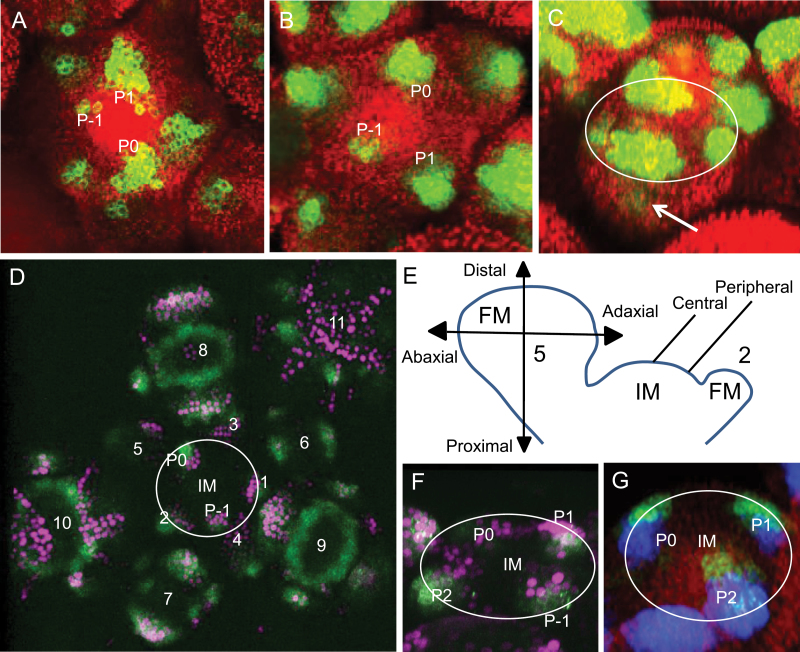
*DR5* and *DRNL* largely mark discrete domains at the IM periphery. (A) *DR5::erGFP* in wild type marks incipient floral anlage and primordia in the IM from P-1. (B) *DRNL::erGFP* in wild type showing expression in bract primordium in P-1 flowers. (C) Wild-type stage-2 flower showing *DRNL* expression of in all four sepals and weak expression in the cryptic bract (marked by an arrow); the circle depicts the boundary of the FM. (D) *DRNL::erGFP* (green), *DR5::NLS3*×*VENUS* (magenta) in a wild-type inflorescence. Note the almost mutually exclusive expression domains except in the tips of the developing sepals and the complete absence of *DR5* expression in the central ring-shaped *DRNL* domain. The IM is marked by a circle. (E) Schematic diagram of an inflorescence showing the central and peripheral IM domains and the FM developmental axes. Numbers represent floral stages according to Smyth et al (1990). (F) An oblique lateral close-up of the same IM as in D, showing differential expression of both markers in incipient and early floral primordia. (G) *DRNL::erCERULEAN* (blue) and *DR5::erGFP* (green) on the flanks of a wild-type IM. Note the separate but partially overlapping domains, with *DR5::erGFP* being higher and more central to the IM than *DRNL::erCERLUEAN*. Red in A, B, C, and G is chlorophyll autofluorescence. The circles in F and G depict the IM boundary. (This figure is available in colour at *JXB* online.)

As the expression patterns of *DR5::GFP* and *DRNL::GFP* are qualitatively similar in the IM (compare Fig. 1A to B), to resolve the relationship between auxin response maxima and cells recruited for lateral primordia marked by *DRNL* within the IM, we raised double transgenic plants that either expressed *DRNL::erGFP* with nuclear-targeted *DR5::VENUS*, or *DRNL::erCERULEAN* with *DR5::erGFP*. Both fluorophore combinations demonstrated a clear difference in the expression domains of *DRNL* and *DR5* at the IM periphery, with that of *DR5* confined to the L1 and slightly higher and more central to the IM centre on the IM flank and that of *DRNL* adjacent at a more peripheral position (Fig. 1F and G) in the L1 and sub-epidermal layers. Analysis of 3D CLSM Z-stack images of both double transgenic lines showed a slight overlap between both expression domains, with some cells at the domain boundaries possibly transiently co-expressing both markers; cells marked by *DRNL* expression at the IM periphery and recruited for lateral organs, thus, largely do not correlate with cells showing an auxin response maximum. Instead, such founder cells in the IM are apparently specified at the outer periphery of such auxin perception maxima. The developmental axes used for descriptions are shown in Fig. 1E. In the IM, the central and peripheral domains are defined, and in the FM the abaxial/adaxial axis is distinguished from the proximodistal axis. In wild type, *DRNL* expression was also observed in the cryptic bract, proximal to the abaxial sepal (Fig. 1C), suggesting that the domain within the IM marked by *DRNL* comprises bract founder cells, those of the abaxial sepal, or a mixture of both. *DR5* expression was mostly associated with organ outgrowth, especially in the margins of the developing sepals (Fig. 1D). No auxin response maxima were present in the ring-shaped domain of *DRNL* expression that prepatterns the medial stamens (Fig. 1D), which is consistent with the hypothesis that *DRNL* marks founder cells earlier than auxin maxima are perceived by the *DR5* promoter.

### *DRNL::GFP* expression in the IM marks bract founder cells

**Fig. 2.**
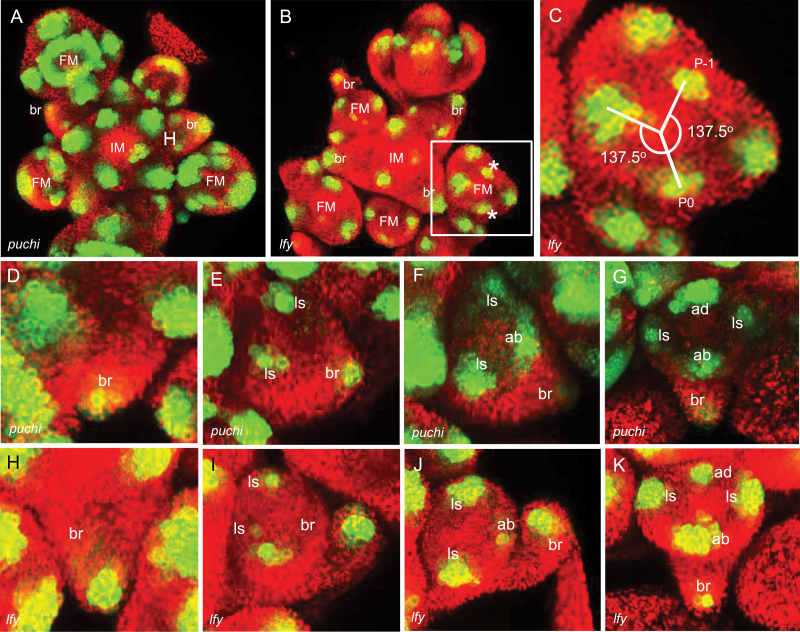
*DRNL* marks bract founder cells and shows an interplay between bract and abaxial sepal initiation in *lfy* and *puchi*. (A) *DRNL::GFP* expression in a *puchi* inflorescence (top view) showing the IM and different floral stages. *DRNL* expression in the IM marks a bract that separates from the IM before sepal initiation. (B) *DRNL::GFP* expression (top view) in a *lfy* inflorescence showing different floral stages. Note bract expression in incipient and early FMs as the bract separates from the IM and elongates. Asterisks mark the continued spiral phyllotaxis of *DRNL* expression within the FM after sepal initiation. (C) Close-up of the inset in B, showing the resumption of a spiral phyllotaxis in the *lfy* IM with a divergence angle of 137.5^o^. (D–K) *DRNL::GFP* marks an elongating bract outgrowing from the IM in D (*puchi*) and H (*lfy*); a bract and two lateral sepals in a lateral oblique view of a stage-2 flower of E (*puchi*) and I (*lfy*); a bract, lateral, and abaxial sepal in a lateral oblique view of a stage-2 flower of F (*puchi*) and J (*lfy*); a bract, lateral, abaxial, and adaxial sepal in a late stage-2 flower of G (*puchi*) and K (*lfy*). Abbreviations: as in Fig. 1 and FM=floral meristem; br=bract. Asterisks show the resumed spiral phyllotaxy of organ initiation in *lfy*. Red represents chlorophyll autofluorescence. (This figure is available in colour at *JXB* online.)

We compared three mutant backgrounds relative to wild type to resolve the identity of cells marked by *DRNL* expression at the IM periphery, which might correspond to a bract, abaxial sepal, or the FM. Strong *DRNL::GFP* expression in the *puchi*, *lfy*, and *ap1 cal* mutant backgrounds indicates that *DRNL* transcription is not under direct control of any of the encoded transcription factors. Characteristic for *lfy* and *puchi* mutants are prominent or small bracts, respectively, which are both marked by *DRNL::GFP* expression in the tips (Fig. 2A, B). However, monitoring *DRNL* expression in *lfy* and *puchi* mutant inflorescences in the time window when the FM separates from the IM revealed that *DRNL::GFP* expression initially marks the bract primordium and not the abaxial sepal primordium, which is initiated subsequently. Extrapolation of the same domain into the IM by comparing expression in multiple flowers at different developmental stages revealed that *DRNL* activity at the IM periphery marks bract founder cells. This is supported by the altered sequence of sepal initiation in *puchi* (Fig. 2D–G) and *lfy* (Fig. 2H–K) flowers: *DRNL* expression in the bract is followed by *DRNL* activity in both lateral sepals, before a GFP signal is detected in the abaxial sepal and finally in the adaxial sepal, instead of the unidirectional sepal initiation in wild type, starting with the abaxial sepal. Following sepal initiation in *lfy*, the lateral and central circular wild-type FM morphogenetic fields of *DRNL* expression are absent, and instead *DRNL* expression foci are arranged spirally (Fig. 2B, C), whereas in *puchi* flowers, *DRNL* expression is whorled, patterns that agree with the different meristem identities, i.e. IM or FM.

**Fig. 3.**
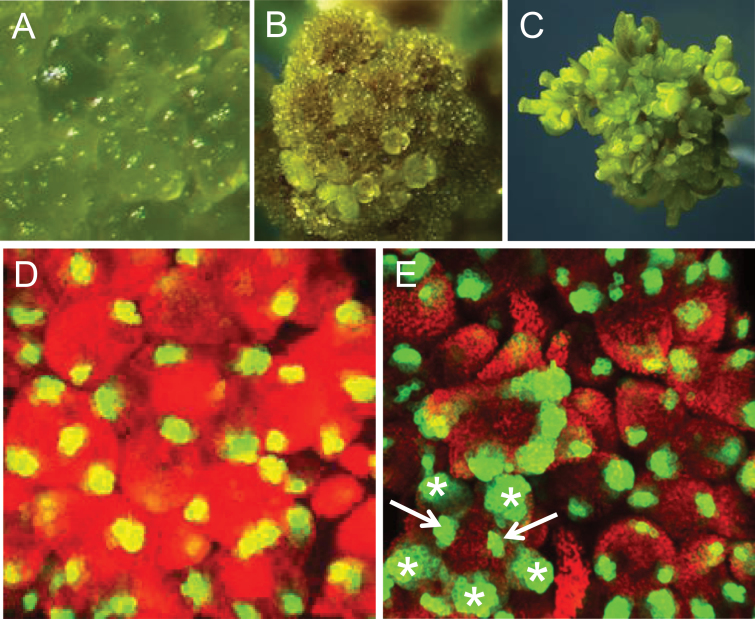
*DRNL::GFP* expression in *ap1 cal* mutants. An *ap1 cal* inflorescence after (A) three weeks, showing only inflorescence meristems (IMs); (B) five weeks, with IMs differentiating into FMs; (C) six weeks, with flowers that develop fertile stamens and carpels. (D) *DRNL::GFP* expression in inflorescences at the same stage as in A, showing bract founder cells. (E) *DRNL::GFP* expression in inflorescences at the same stage as in B, showing stamen and carpel founder cells. Note the absence of broader expression domains such as a central ring-shaped field. Stamen and carpel founder cells are marked by asterisks and arrows, respectively. Red in D and E represents chlorophyll autofluorescence. (This figure is available in colour at *JXB* online.)

Further evidence that *DRNL* expression foci in the IM mark cells that have not acquired floral meristem identity is their presence in young *ap1 cal* mutant inflorescences in the absence of differentiated FMs (Fig. 3A, D). The presence of a FM phyllotaxy implies that *DRNL* expression marks a cellular identity that might represent bracts and precedes FM and sepal initiation. Subsequently, when the IMs of *ap1 cal* plants produce FMs and differentiate *ap1*-like floral organs (Fig. 3B and C), *DRNL* marks stamens and carpels (Fig. 3E, marked by asterisks and arrows). Imaging of *DRNL::GFP* in three mutant backgrounds, therefore, shows that cells recruited for new lateral primordia at the IM periphery are not specified to acquire sepal identity, as exemplified in the *puchi* or *lfy* background, and neither have FM identity in the *ap1 cal* double mutant. All three mutant backgrounds affect cell fate in new lateral branches, but bracts subtending *puchi* and *lfy* flowers cause an altered sequence of sepal initiation, with the two lateral sepals following the medial ones in a decussate phyllotaxy. A reiteration of a decussate patterning mode is evident in the two lateral morphogenetic fields of *DRNL::GFP* expression for petals and lateral stamens in bilaterally symmetrical stage-3 flowers.

### Pentamery in the *pan-1* mutant is initially unidirectional and subsequently centripetal

**Fig. 4.**
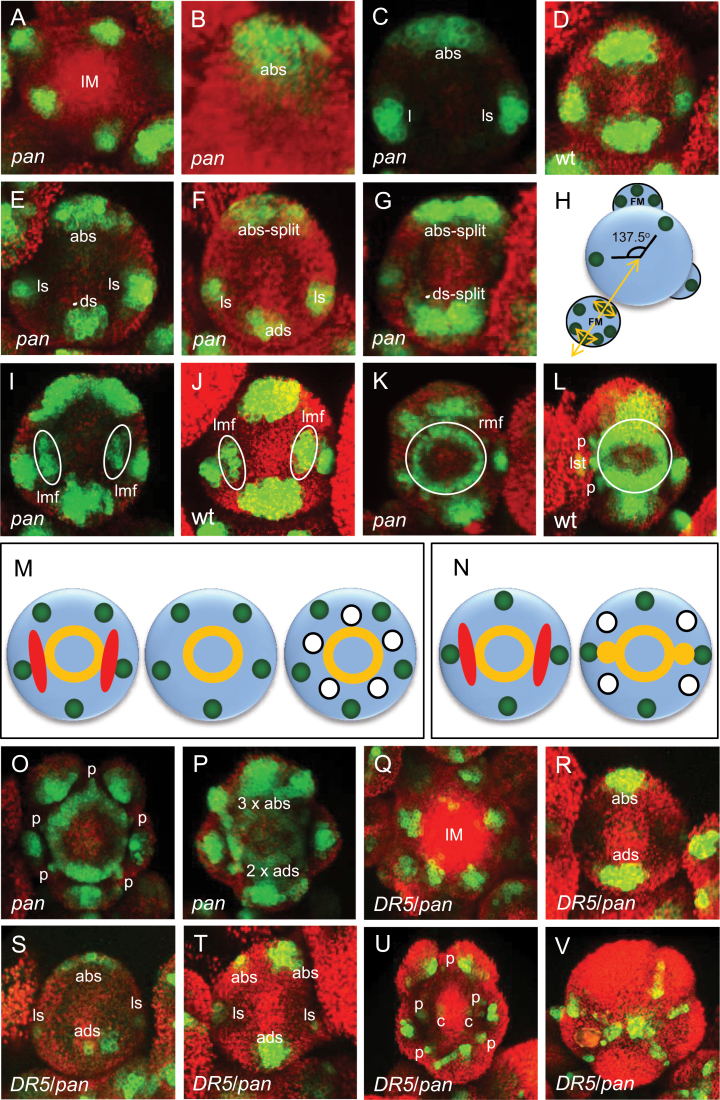
Expression of *DRNL::GFP* and *DR5* in *pan-1* and wild type throughout floral development: (A) *DRNL::GFP* expression in the *pan* IM periphery; in an early stage-2 *pan* flower marking (B) the abaxial sepal; (C) the abaxial and two lateral sepals; (D) *DRNL::GFP* expression marking all four sepals in a wild-type stage-2 flower; (E) marking all sepals in a stage-2 *pan* flower (note the broadening of the abaxial domain); (F) in a late stage-2 *pan* flower showing the splitting of the abaxial sepal founder cell population into two discrete foci; (G) in a late stage-2 *pan* flower showing the splitting of the abaxial and adaxial sepal founder cell populations; (H) schematic diagram showing wild-type sequence of sepal initiation (green circles) as the FM separates from the IM, and the splitting of sepal founder cells in lateral directions from the abaxial and adaxial domains, along an abaxial/adaxial axis from the centre of the IM (yellow arrows); *DRNL::GFP* expression showing the two lateral expression domains inner to the sepal domain in (I) a stage-3 *pan*; (J) a stage-3 wild-type flower; (K) an early stage-4 *pan* flower showing the central ring-shaped domain (note the absence of petal or stamen founder cells); (L) a stage-4 wild-type flower, showing the central ring-shaped domain and the resolution of the lateral domains each into founder cells of two petals and one lateral stamen. (M) A schematic representation of *DRNL* expression in floral organ founder cells in the outer three whorls in stage-3 and 4 *pan* flowers, showing sepals in green, the lateral domains that subsequently disappear (red), the central ring-shaped domain (yellow) that will produce five radial stamens, and the initiation of five petals in a radial pattern interstitially between the sepals. (N) wild-type flowers, showing the sepals (green), the red lateral domains that each resolves into two petals, and a single lateral stamen domain and the central yellow ring-shaped domain that will give rise to the medial stamens. The location of the IM is to the bottom of each image. *DRNL::GFP* in (O) a late stage-4 *pan* flower (note the initiation of petal founder cells interstitially between the sepals); (P) a stage-4 *pan* flower showing splitting of the abaxial sepal founder cells into three and the adaxial into two petal founder cells, and the fragmentation of the ring-shaped domain into stamen founder cells. *DR5::GFP* expression in *pan* in (Q) the IM periphery; (R) the abaxial and adaxial sepals of a stage-2 flower; (S) all sepals in a stage-2 flower (note the broadening of the abaxial domain); (T) a late stage-2 flower after bifurcation of the abaxial sepal domain; (U) a stage-5 flower showing petal founder cells interstitially between the sepal primordia; (V) a stage-6 flower showing strong expression associated with the margins of the expanding sepals. Abbreviations: IM=inflorescence meristem; ab=abaxial sepal; ad=adaxial sepal; ls=lateral sepal; lmf=lateral morphogenetic field; p=petal; rmf=ring-shaped morphogenetic field; c=carpel. Red represents chlorophyll autofluorescence. (This figure is available in colour at *JXB* online.)

The *pan* mutant has a mostly pentameric arrangement of floral organs at least in the first two whorls, giving a flower with a radially symmetrical perianth (Table 1). To address the origin of the ectopic perianth organs and to monitor early *pan* floral development, expression of *DRNL::GFP* in the *pan-1* background was monitored throughout ontogeny by confocal microscopy. The analysis of multiple individual inflorescence shoots showed that the qualitative pattern of *DRNL* expression is initially conserved compared with wild type (Chandler *et al.*, 2011); *DRNL* is expressed in the IM (Fig. 4A) and marks sepal founder cells in stage 2 and 3 *pan* flowers in the order: abaxial sepal, both lateral sepals simultaneously, adaxial sepal (Fig. 4B, C and E), in the same sequential order as wild type (Fig. 4D and Chandler *et al.* 2011). However, starting in floral stage 2, the abaxial sepal founder cell population becomes laterally broader and seems to divide into two or three distinct *DRNL* expression foci (245 from 287 *pan* flowers; Fig. 4E, F, G, P). This can also occur for the adaxial sepal domain (Fig. 4G, P), at a lower frequency (42 from 287 flowers analysed). Division of both domains can give rise to up to seven sepal primordia (Fig. 4H, P). Although it cannot be ruled out that splitting of lateral sepals also occurs, this was not unequivocally observed among the inflorescences analysed. Furthermore, the derivation of ectopic sepals can be inferred from their spacing in 3D images, which show that the buttresses of the ectopic sepals are almost always more distal than the lateral ones, supporting their preferential origin from abaxial or adaxial sepal founder cell populations. The initiation of ectopic sepals in the *pan* mutant subsequent to *DRNL* sepal founder cell expression suggests that *PAN* functions temporally later than *DRNL* in flower development.

**Table 1.** Floral organ numbers for wild type and various mutants and mutant combinations

Genotype	Sepals	Petals	Lateral stamens	Medial stamens	Carpels
*prs*	3.60±0.66	4.03±0.22	1.39±0.60	3.99±0.10	2.14±0.35
*prs drnl*	3.91±0.31***	4.00±0.21	0.86±0.71***	3.94±0.23*	2.00±0.00***
*drnl*	4.00±0.00	3.99±0.01	0.66±0.76	3.97±0.17	2.00±0.00
*pan-1*	5.20±0.47	4.97±0.41	5.74±0.73		2.08±0.27
*pan-1 drnl*	4.47±0.66***	4.28±0.68***	4.16±0.79***		1.83±0.38***
*pan-1 prs*	4.09±0.40***	4.25±0.54***	5.72±0.51		2.00±0.00*
Col	4.00±0.00	4.00±0.00	1.80±0.49	4.00±0.00	2.00±0.00
L*er*	4.02±0.14	4.04±0.20	1.86±0.45	4.04±0.20	2.00±0.00

Values are means±SD (*n*=100). For single and double mutants, including *pan-1*, the total stamen number is given, owing to the inability to distinguish between lateral and medial stamens in a radial arrangement. Values marked with asterisks for *pan-1 drnl* or *pan-1 prs* are significantly different from those from *pan-1*; values for *prs drnl* are compared with those of *prs*; *P<0.05; **P<0.001: ***P<0.0001.

According to *DRNL* expression, petal founder cells (four to six) appear in the *pan* interstitial sepal domains before the resolution of the central ring-shaped morphogenetic field into four to seven stamen founder cell foci in a radial pattern (Fig. 4O). Both lateral morphogenetic fields that in wild type mark the lateral stamens and petals, and the ring field that pre-patterns medial stamens are also conserved in *pan* (Fig. 4I to L). However, in contrast to wild-type flowers, *pan* petal and lateral stamen founder cells do not arise from the resolution of two lateral domains prepatterned by *DRNL::GFP*, which are also present in *pan* mutant flowers but disappear slightly later (Fig.4K). These differences between wild-type and *pan-1* mutant flowers are schematically depicted in Fig. 4M, N. Although the number of sepals and petals in individual *pan-1* flowers varied between 4–7 and 4–6, respectively, the number of petals was highly significantly correlated with that of sepals (Pearson’s *r*=0.34; *P*<0.001).

To correlate ectopic sepal formation in the *pan* mutant with auxin response maxima, we used the *DR5::GFP* reporter, which sequentially marks the initiation of most floral organs in the *pan-1* mutant background: in stage-1 and 2 flowers, *DR5::GFP* is expressed in the IM (Fig. 4Q) and then appears simultaneously in both the abaxial and the adaxial sepals at stage 2 (Fig. 4R). Similar to the *DRNL::GFP* marker, subsequent expression in stage 2 often gives rise to a broad domain in the abaxial (Fig. 4S) or adaxial sepal domain that separates into two or more foci later in stage 2, to mark ectopic sepals (Fig. 4T). However, in contrast to the serial order of *DRNL* expression in the lateral and adaxial sepals of early stage-2 flowers, the *DR5*-derived signals appear with the onset of morphogenesis in the lateral sepals at the end of stage 2. Similar to *DRNL::GFP*, *DR5::GFP* activity marks petal primordia at stage 4 (Fig. 4U), alternating between the outgrowing sepals. However, in *pan-1*, although *DR5* marks carpel primordia at stage 4 (Fig. 4U), little or no *DR5* expression is associated with stamen initiation. During later floral development, *DR5* expression is also strongly associated with organ outgrowth and marginal growth, especially of the sepals, as shown in Fig. 4V.

### Elaboration of the abaxial/adaxial floral body plan is partially dependent on *PRS/WOX3* function

**Fig. 5.**
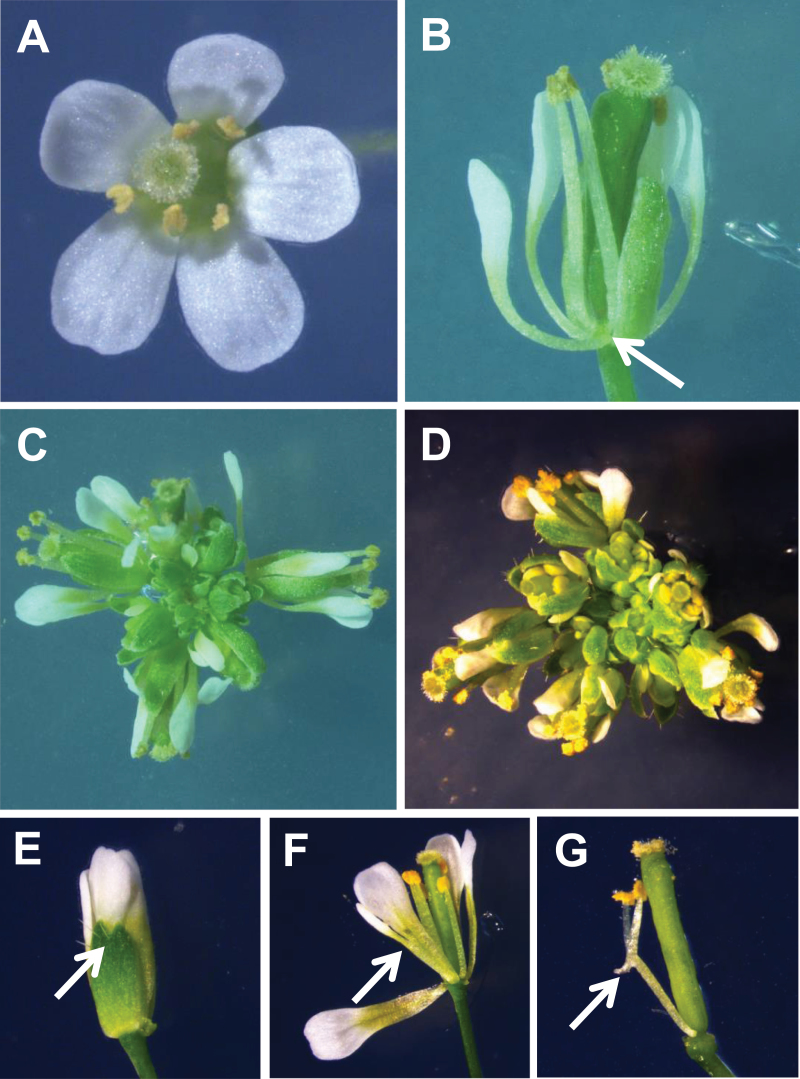
Phenotype of *pan-1*, *prs* and *pan-1 prs* double mutants. (A) *pan-1* flowers; (B) *prs* flowers. Note the absence of the lateral sepal domain, marked by an arrow. (C) a *prs* inflorescence; (D) a *pan-1 prs* inflorescence. Fusion in *pan-1* (marked with arrows) of (E) sepals; (F) petals and (G) stamens. (This figure is available in colour at *JXB* online.)

To investigate the genetic interaction between mutants that show either a pronounced radial (*pan-1*) or an adaxial–abaxial (*prs*) floral body plan, both mutants were crossed. Plants in the F_2_ population showed only wild-type, *pan-1*, or *prs* single mutant phenotypes and no additive or synergistic phenotypes, suggesting that one mutation might be epistatic over the other. To resolve this, double mutant plants were identified by selecting plants with a *prs* mutant phenotype and identifying individuals that were also homozygous for *pan*, by generating PCR amplicons spanning the nucleotide insertion after base 468 in the *pan-1* mutant ORF (Chuang *et al.*, 1999) followed by sequencing. Single *pan-1* mutants have a mostly pentameric arrangement of sepals and petals and between five and six stamens (Fig. 5A; Table 1; Running and Meyerowitz, 1996), whereas *prs* mutants have a reduction in whorl one organs (Fig. 5B), an occasional overproliferation of carpel valves (Table 1; Matsumoto and Okada, 2001), and an open-flower phenotype (Fig. 5C). Double *pan-1 prs* mutants appeared more similar to *prs* single mutants in terms of the open flower phenotype (Fig. 5D) and organ numbers were more similar to those of *prs* in all floral whorls except for stamens, which were not significantly different in number from those in *pan-1* alone (Table 1). This genetic interaction suggests that ectopic sepals and petals in *pan* are partly dependent on *PRS* function or that pathways involving both genes merge. Additionally, for both *prs drnl-1* and *pan-1 drnl-1* double mutants, the number of all organs except for petals of *prs drnl* was significantly closer to that of *drnl* mutants than to either *pan-1* or *prs* single mutants (Table 1), suggesting that both *PAN* and *PRS* functions in some whorls genetically interact with *DRNL* function. The splitting of sepal founder cell populations to create ectopic sepals is reflected phenotypically in the *pan* mutant by the occasional fusion of sepals (4.5%, *n*=335, Fig. 5E). Fusion of petals (1.5%, *n*=335, Fig. 5F) and stamens (0.3%, *n*=335, Fig. 2G) also occurred, preferentially at the abaxial position and presumably owing to imprecise resolution of the *DRNL* morphogenetic field and the generation of primordia in close proximity.

## Discussion

Confocal imaging of *DRNL* at high spatial and temporal resolution in several different mutant backgrounds reveals novel aspects of early floral morphogenesis, delineates an early abaxial/adaxial polarity that determines sepal initiation, and implicates an alternative cell proliferation mechanism to that involving *WUS/CLV3* before the reestablishment of a centripetal organogenesis mode for inner whorl organs.

### *DRNL* and auxin response mark a lateral organ phytomer within the IM

One of the most important findings is that *DRNL* expression foci in the IM do not correlate with auxin response maxima, thought to pre-pattern FM initiation (Reinhardt *et al.*, 2003, Heisler *et al.*, 2005). The *DRNL* and *DR5* expression domains both agree with the incipient FM phylotactic pattern, but are spatially distinct, with auxin response maxima at a more central position in the IM, and *DRNL* expressed more to the IM periphery; possibly both domains transiently overlap at their common border. Accordingly, cells recruited for the bract/abaxial sepal in wild type or the bract in *puchi* and *lfy* mutants, which are marked by *DRNL* expression at the IM periphery, are not prepatterned by canonical response maxima. Although the lack of *DR5* expression does not exclude lower threshold or non-canonical responses, this finding is consistent with the dispensability of canonical AuxREs in the *DRNL* promoter (Chandler *et al.*, 2011). Despite spatial differences, *DR5* and *DRNL* expression appear simultaneously at the IM periphery and here, as in Heisler *et al.*, (2005), the appearance of auxin response maxima correlates with the formation of incipient P-1 primordia in the appropriate phylotactic pattern.

Early FM development is poorly understood (Chandler, 2012); the earliest oriented cell divisions in the IM occur along a plane parallel to the axis of primordium outgrowth and these cell divisions suggest that FM fate is established between P-1 and P0 (Reddy *et al.*, 2004). This is at variance with the previously proposed arrangement of four FM founder cells in a block arrangement in the IM that divide to give a concentric group of cells (Bossinger and Smyth, 1996). In both *puchi* and *lfy* mutants, *DRNL* expression clearly marks the bract as it separates from the IM and persists in the abaxial bract tip as it outgrows. Conforming to phytomer theory, FMs are considered to initiate in the axil of cryptic *Arabidopsis* bracts (Kwiatkowska, 2006; Alvarez-Buylla *et al.*, 2010), their development being suppressed in wild type but elaborated in *puchi* and *lfy* mutant backgrounds. The up-regulation of *AINTEGUMENTA* and down-regulation of *SHOOTMERISTEMLESS* (*STM*) expression can act as markers for the cryptic bract region (Long and Barton, 2000) and *STM* expression is completely absent in P0 FM primordia, where *DRNL* expression is strong in the wild-type IM, and has been previously related to the specification of the abaxial sepal (Chandler *et al.*, 2011). Bifurcation of this *DRNL* expression domain into two foci—an apical and a subtending basal one (Chandler *et al.*, 2011 and here)—suggests that in stage-2 wild-type flowers *DRNL* expression distally marks the tip of the abaxial sepal and transiently persists in the proximal subtending cryptic bract region, which was never observed in *lfy* and *puchi* IMs, where *DRNL* expression is confined to the apical tip of the emerging bract. Whereas the *LFY* expression domain is larger than the FM anlage (Grandjean *et al.*, 2004), the *PUCHI* transcription domain does not overlap with the cryptic bract domain (Karim *et al.*, 2009), but independently of whether a bract develops or is suppressed, the activity of the *DR5* and *DRNL* promoters provides molecular evidence that two groups of cells with distinct identities coexist in incipient floral phytomer domains at the IM periphery: the auxin response maximum oriented towards the IM centre, and the *DRNL* expression domain more peripherally; a slight overlap of the *DRNL* and *DR5* domains might mark a subset of cells with mixed or special identity.

Later in development, the discrepancy between auxin response and founder cell populations marked by *DRNL* within the FM questions the role of auxin in floral organ founder cell specification and the conclusion that *DRNL* precedes *DR5* expression in some whorls (Chandler *et al.*, 2011) has been more fully addressed here using double transgenic lines with two sets of *DRNL*/*DR5* fluorophores. In wild type, sequential sepal initiation is not coincident with canonical auxin response maxima, which appear in the abaxial and adaxial sepals first, which are the first sepals to emerge, followed by the two lateral sepals (van Mourik *et al*., 2012). In stages 3 and 4, there is almost no coincidence of expression between *DRNL* and *DR5*; expression of *DR5* is absent from the central ring-shaped morphogenetic field of *DRNL* expression, but is strong in the sepal margins, suggesting that auxin responses are more related to organ outgrowth (Aloni *et al.*, 2006). Clearly, different organs have different auxin response requirements, different threshold auxin responses, or non-canonical responses.

### The interplay between bract and abaxial sepal development in *Arabidopsis*

An interesting question with regard to development of the floral primordium is whether the central/peripheral information in the IM relates to the abaxial/adaxial polarity within the floral bud. Wild-type sepal founder cells are recruited unidirectionally in the sequential order: abaxial, two lateral, and adaxial (Chandler *et al.*, 2011). However, when the bract is elaborated in *lfy* or *puchi* flowers, two lateral sepals are initiated before the abaxial and adaxial sepals. As ablation of the LFY domain in wild type resulted in flowerless bracts (Nilsson *et al.*, 1998), it had been assumed that bract development is suppressed by signals from the floral bud, or alternatively that ablation occurred after founder cells for a new lateral primordium were specified at the IM periphery and only interfered with subsequent FM elaboration. The altered series of events is compatible with both assumptions: in the absence of *LFY* function, founder cells at the IM periphery acquire bract identity, whereas specification of four sepals in the absence of *CLV3/WUS* activity indicates that a functional FM is elaborated secondarily.

Bract suppression, at least in the Cruciferae, is considered a derived trait (Penin, 2008) that has arisen independently in various plant radiations (Whipple *et al.*, 2010). Accordingly, it has been suggested that *LFY* and *PUCHI* suppress bract morphogenesis (Coen and Nugent, 1994; Karim *et al.*, 2009). Alternatively, they might promote the recruitment of bract founder cells for specification of the abaxial sepal, so that their absence of function results by default in a bract. The interplay of founder cell recruitment between the two organs is compatible with the absence of *PUCHI* expression in the prospective bract region (Karim *et al.*, 2009) but with its early activity within the floral bud, where the abaxial bract is suppressed in wild type but specified in *puchi* or *lfy* mutant flowers. The separation of the cryptic bract domain into a short-lived proximal and a more persistent distal domain in wild type (Chandler *et al.*, 2011) indicates that cell identity might not be specified at the IM periphery, but depends on fine-tuning through *LFY* and *PUCHI* functions. The default bract, i.e. leaf-like identity of lateral organ founder cells at the IM periphery, is consistent with the spiral phyllotaxy of *DRNL::GFP* expression foci in *ap1 cal* IMs, which correlates with branch points of new floral phytomers. The *ap1 cal* inflorescences at this stage either reiterate IMs instead of FMs (Bowman *et al.*, 1993; Ferrándiz *et al.*, 2000) or alternatively might represent FMs that are arrested at an early stage that precedes the morphological appearance of floral organ primordia, and cells expressing *DRNL::GFP* have either thus lost IM identity, but have not acquired FM identity or might show some early floral characteristics.

Other species than *Arabidopsis*, such as sunflower, have competing allocations of cells between the bract and FM; the floret bract and FM are initiated simultaneously and signals along an abaxial/adaxial gradient are necessary for floret primordium bifurcation (Fambrini *et al.*, 2003). Mutation of the *MISSING FLOWERS* gene disrupts this gradient and causes abaxial floral fate. Similarly, in *Calendula officianalis*, bracts are subsequently absent and solely floret fate results (Fambrini *et al.*, 2003). The mechanism of this interplay between founder cell populations remains unknown, but might be similar to the bifurcation that occurs of common primordia in legumes (Ferrándiz *et al.*, 1999; Benlloch *et al.*, 2003) to generate distinct stamen and petal founder cell populations.

### *PERIANTHIA* implements outer whorl abaxial/adaxial polarity by restricting organ number

The conservation of early *DRNL* expression in *pan* compared with wild type includes the sequential appearance of sepals, the two lateral morphogenetic fields at floral stage 3, and the ring-shaped central morphogenetic field. However, bifurcation of abaxial and adaxial sepal founder cell groups to create ectopic sepals is an important novel description of the *pan* phenotype and demonstrates that wild-type *PAN* function restricts organ initiation and exerts a preference for the medial sepals. Splitting of the sepal founder cell domains in the *pan* mutant subsequent to *DRNL* expression places *PAN* function temporally later than *DRNL*. We did not co-monitor *DRNL* and *DR5* expression in the *pan-1* background, but the temporal and spatial correlation between the *DR5* and *DRNL* expression domains in stage-2 flowers by comparing expression in individual flowers at similar developmental stages, suggests that this detached organogenesis process involves canonical auxin responses.

Although constantly active in the IM centre, the stem cell markers *CLAVATA3* and *WUSCHEL* and the meristem marker *STM* are absent during early FM development until floral stage 2 when they re-establish and mark a stem-cell population and meristematic cells in the FM (Long *et al.*, 1996; Goldschmidt *et al.*, 2008; Yadav *et al.*, 2009). Accordingly, sepals are initiated and split in the *pan* mutant in the absence of a central FM stem cell population and alternative cell proliferation mechanisms have to provide cells for recruitment. The genetic interaction between *prs* and *pan* suggests that the *pan* phenotype is partially dependent on *PRS* function and thus that tetramery might predominate over pentamery in the perianth. *PRS* is a *WUSCHEL-related homeobox* (*WOX*) gene that regulates cell proliferation and organogenesis in the lateral domains of stage-1 floral primordia and of the abaxial and axial sepals (Matsumoto and Okada, 2001). The *prs* mutant phenotype is fully complemented by *WUS* expression from the *PRS* promoter (Shimizu *et al.*, 2009); thus, *WUS* and *PRS* genes encode similar protein functions but substantially differ in their transcription pattern. In the absence of *WUS* expression, the *prs* phenotype and the genetic interaction between *pan* and *prs* imply that the early floral initiation phase of *pan* implements cell proliferation potential provided by *PRS*. There is a correlation in *prs* mutants between the absence of stipules in the vegetative phase and lateral sepals in the flower (Nardmann *et al.*, 2004). *PRS* acts redundantly with *WOX1* in the leaf middle domain to integrate adaxial/abaxial positional cues for lamina expansion in *Arabidopsis* (Nakata *et al.*, 2012) or *Petunia* (Vandenbussche *et al.*, 2009). These observations suggest that the *PRS* regulation of lateral domains in flowers represents a conserved cell proliferation function in leaves. Although preferentially confined to lateral FM domains, *PRS* is also present in the abaxial domain of stage-1 flowers (Matsumoto and Okada, 2001), where it might regulate cell proliferation in the abaxial sepal region. In addition, the *WOX1* orthologues *STENOFOLIA* in *M. truncatula* or *LAM1* in tobacco affect auxin levels (Tadege *et al.*, 2011) and *PAN* also functions upstream of auxin responses by targeting *YUC1* and *YUC4* (Maier *et al.*, 2011). The convergence of the *PRS* and *PAN* pathways on auxin might be another explanation why the absence of *PRS* affects the medial sepals.

Elevated *STM* expression in *pan* mutant stage-1 floral buds relative to wild type (Maier *et al.*, 2011) supports an increase in meristematic activity, which might contribute to the splitting potential of abaxial and adaxial sepal domains. Genetically, we show here that *PAN* and *PRS* functions also interact with those of *DRNL*, which is compatible with their transcription patterns: *PAN* is expressed throughout the IM and FM during initiation of all floral organs (Chuang *et al.*, 1999) and thus overlaps with *DRNL* in floral organ founder cells, whereas *DRNL* expression is unaltered in *prs* mutant flowers (Chandler *et al.*, 2011).

### Initiation of inner whorl organs: the transition from an abaxial/adaxial polarity to a centripetal mode

In contrast to sepals, the specification of petal primordia occurs synchronously, but reflects the number and position of outer whorl organs in wild-type and *pan* mutant flowers. Wild-type petal initiation is dependent on auxin biosynthesis and signalling in the intersepal zone. Alteration of the intersepal distance through the *PETAL LOSS* transcription factor disrupts these signals and reduces petal number (Lampugnani *et al.*, 2013). Thus, petal number correlates with that of sepals, although sepals are initiated sequentially in wild type or are in part derived from sepal founder cell population bifurcation along an abaxial/adaxial axis in *pan* mutant flowers. There is a significant correlation between sepal and petal number in the *pan* mutant, which also suggests that ectopic petal initiation is dependent on signals from ectopic sepals.

Linkage among perianth organ initiation identifies a transitioning between organogenesis signals and defines a hierarchy of positional signals. The re-emergence of a stem cell population in the FM centre at floral stage 2 is a key event, and one hypothesis is that it establishes a centripetal organ initiation mode for subsequent organ whorls. This is supported by a particular sequential order of outer whorl organ initiation in the *lfy* mutant, even if this is not unidirectional, followed by the re-establishment of spiral phyllotaxis of *DRNL* expression. Wild-type medial stamens and carpels are clearly initiated centripetally from the resolution of a ring-shaped morphogenetic field and as final central organs, respectively, and we argue that petals and lateral stamens are also initiated centripetally from stage 3 onwards, after unidirectional sepal initiation. *WUS* function is dispensable for petal initiation, as mean petal number is only slightly altered by loss of *WUS* function (Laux *et al.*, 1996). However, *WUS* affects meristem size and is not an organ specifier. The dependency of other petal specifiers such as *PETAL LOSS* on sepals shows that petals in wild type are specified independently to all stamens and before them. Lateral stamens and petals are prepatterned by bilaterally symmetrical lateral *DRNL* domains of expression and medial stamens by a ring-shaped *DRNL* domain. However, specification signals, which are mostly unknown, but for petals depend on sepals, are responsible for the resolution of the lateral and medial morphogenetic fields in whorls 2 and 3. Thus, centripetal signals that superimpose onto the *DRNL* morphogenetic fields are still compatible with a centripetal floral patterning mode. Furthermore, the lateral morphogenetic fields that appear synchronously and bilaterally at stage 3 in *pan* flowers are not elaborated for petal and lateral stamen initiation, possibly because the instructive character or space constraints of the outer whorl organs overwrite the endogenous blueprint and cause supernumerary organs in the inner whorls. Patterning of the *Arabidopsis* wild-type floral body plan thus begins with unidirectional polarity and bilateral symmetry for sepals and we posit that it subsequently transitions to a centripetal mode for inner whorl organs. However, the testing of this hypothesis is dependent on further research that identifies the individual specification signals for inner organs. Pentamery represents the ancestral floral body form (Endress, 2001), suggesting that the acquisition of *PAN* might have contributed to the evolution of tetramous flowers as in the Brassicaceae. *PAN* neither alters polarity nor symmetry of the early floral patterning programme, but controls splitting of founder cell populations in the outer whorl, before the transition from an initially abaxial/adaxial polar ground state to an autonomously acting floral meristem, with *WUS* and *CLV3* controlling stem cell homeostasis.

### Conclusions and a model of FM initiation and patterning

**Fig. 6.**
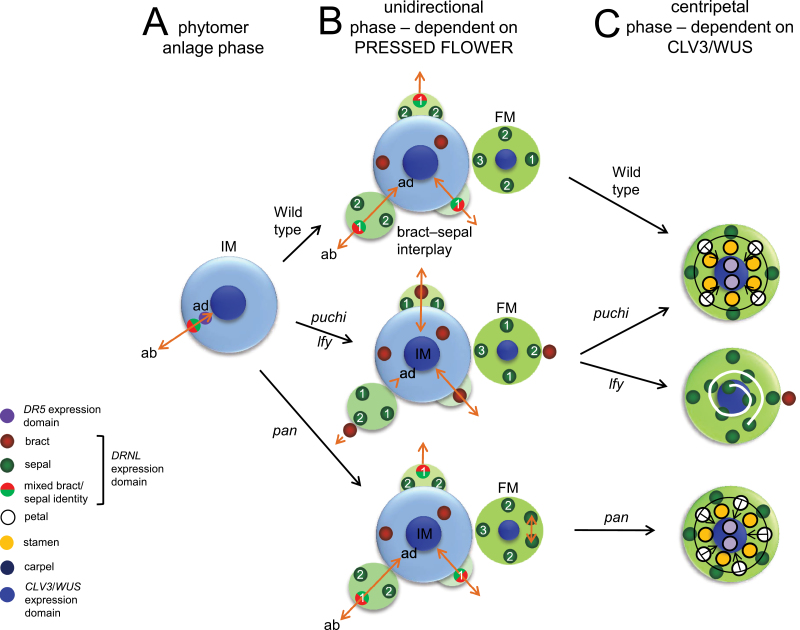
Model summarizing three transitioning phases of *Arabidopsis* FM development: (A) phytomer establishment phase when the IM initiates discrete *DRNL* and *DR5* expression foci at the periphery. Bract initiation establishes abaxial and adaxial polarity and there is interplay between the bract and the FM for founder cells. (B) The unidirectional phase during stage-1 and 2 that coincides with the absence of a stem cell population and the sequential and unidirectional initiation of sepals is dependent on PRESSED FLOWER function. Numbers show the order of sepal initiation for wild-type flowers (above) and *lfy* and *puchi* mutants (centre) and *pan* mutants (below). (C) The centripetal phase is associated with the re-establishment of a stem cell population in the FM centre from late stage-2, and initiation of petals, stamens, and carpels in concentric whorls (shown by the black circle with arrows).

The major conclusions are that FM organ initiation in *Arabidopsis* occurs via two alternative mechanisms. The first involves the establishment of abaxial/adaxial polarity within a narrow temporal window by the specification of bract founder cells at the IM periphery as marked by *DRNL*, independently from canonical auxin response maxima. Conforming to phytomer theory, the concomitant establishment of transient auxin response maxima in adjacent more central IM domains possibly prepatterns FM initiation in the bract axil (Fig. 6A). Subsequent initiation of the abaxial sepal is dependent on bract suppression, presumably owing to an interplay for founder cell recruitment between competing identities (Fig. 6.B). Sequential and unidirectional sepal initiation along the abaxial/adaxial axis can be modulated by *PAN* and is dependent on *PRS*. Furthermore, bract outgrowth alters the sequence of sepal initiation in *lfy* and *puchi* (Fig. 6B). Following re-establishment of the stem-cell zone in the FM centre, transition to a centripetal mode of organ initiation occurs, with petals, and lateral and medial stamens arising from two types of morphogenetic fields marked by *DRNL*, that concentrically elaborate the inner organ whorls, except for in *lfy*, where a spiral phyllotaxy is re-established (Fig. 6C).

Biphasic floral organ initiation that transitions from a unidirectional axis to a centripetal mechanism has implications for comparative evolutionary developmental biology, including whether radial floral body plans derive from an initial abaxial/adaxial polarity. Heterochrony of either phase or the regulation of sepal meristy by *PAN* would represent one way to modulate body plan development and might contribute towards the variation in floral morphology observed in plants, especially in species that display marked unidirectional organ initiation. Only the final step of organ differentiation has been subjected to an Evo-Devo comparison based on ABCE organ identity genes; however, diversity of floral architecture, i.e. the number, position, fusion, or separation of floral organs, has been a major source of evolutionary adaptation. Polarity in the early patterning programme, its dependence on an alternative cell proliferation pathway and its instructive character for the inner whorls when the FM has acquired autonomy has neither been considered nor has been apparent based on morphological criteria.

## Author contributions

JWC developed the concept, performed the experiments, and wrote the manuscript; WW commented on the manuscript, provided useful discussions, and marker lines.
